# Supplementary material for: Pathogenic tau induces an adaptive elevation in mRNA translation rate at early stages of disease
Source: Aging Cell. 2024 Jun 26;23(10):e14245. doi: 10.1111/acel.14245 (PMC11464109; doi:10.1111/acel.14245)
Supplement: Supplementary file 1 — Data S1: [file ACEL-23-e14245-s001.docx]

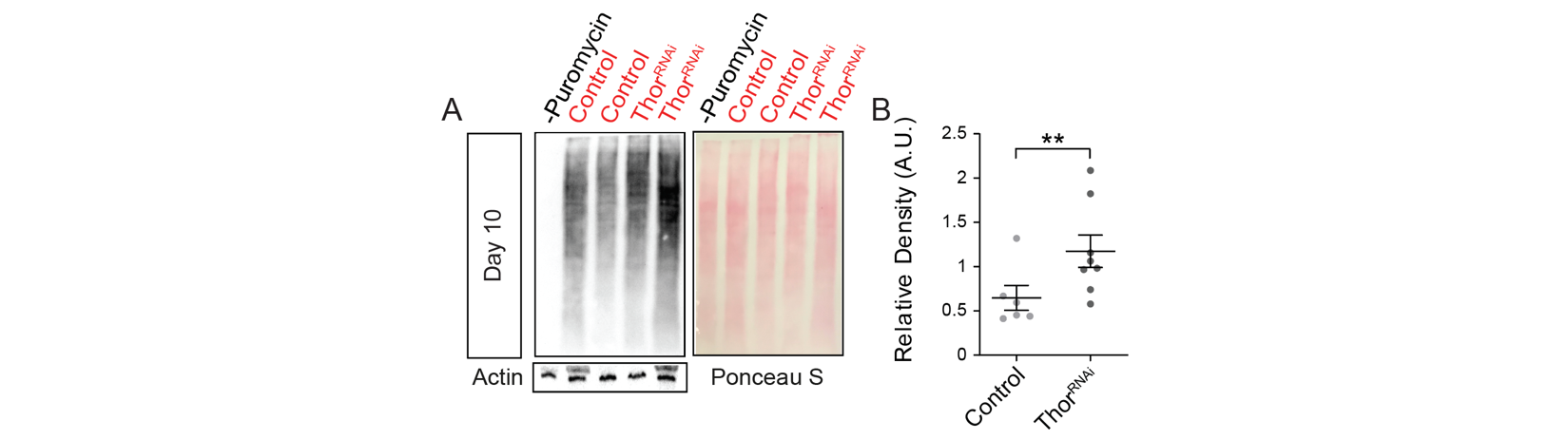
**Supplemental Figure 1 | Derepression of mRNA translation significantly increases puromycin staining by western blot. A)** Genetic reduction of Thor, *Drosophila* homologue of eIF4E binding protein, specifically in neurons of *Drosophila* fed 10 mM puromycin for 24 hours significantly increases puromycin staining by western blot relative to control, with quantification (B). p**<0.01, unpaired t-test. Error bars=SEM.

| **Name** | **Full Genotype** | **Insertion Chromosome** | **Stock #** | **Source** |
| --- | --- | --- | --- | --- |
| Upf1^RNAi^ | y[1] v[1]; P{y[+t7.7] v[+t1.8]=TRiP.GL01485}attP2 | 3 | 43144 | Bloomington (DRSC-TRiP) |
| Upf1^OE^ | w[*]; P{w[+mC]=UASp-GFP.Upf1}2 | 2 | 24623 | Bloomington |
| Upf2^OE^ | w[*]; P{w[+mC]=UAS-Upf2.A}3 | 3 | 60578 | Bloomington |
| UAS-tau^R406W^ | UAS-tau^R406W^ | 3 |  | Mel Feany  (Wittman et al., 2001) |
| UAS-tau^WT^ | UAS-tau^WT^ | 3 |  | Mel Feany  (Wittman et al., 2001) |
| UAS-SCA3 | P{UAS-SCA3.fl-Q84.myc}7.2 | 3 |  | Nancy Bonini  (Warrick et al., 1998) |

**Supplemental Table 1 |** Name of *Drosophila* lines as referred to in paper as well as full genotype, insertion chromosome, stock number and source. Bloomington (Bloomington *Drosophila* Stock Center).

| **Antibody** | **Host** | **Western** | **IF** | **Source** |
| --- | --- | --- | --- | --- |
| cTau | Mouse | 1:100,000 |  | Dako # A0024 |
| LaminDmO | Rabbit |  | 1:200 | Paul Fisher (R836) |
| Puromycin [3RH11] | Mouse | 1:1,000 | 1:50 | Kerafast, #EQ0001 |
| Rps3 | Rabbit | 1:1,000 |  | Bethyl laboratories # A303-840A |

**Supplemental Table 2 |** Antibodies used in immunofluorescence (IF) and western blotting.

| **Gene** | **Human ortholog** | **Function** | **AD-association** | **Causes neurodeg-eneration** | **mRNA/**  **protein levels in AD** | **Neuroprotective in AD** | **Translation efficiency** |
| --- | --- | --- | --- | --- | --- | --- | --- |
| Breathless | FGFR1 and FGFR3 | Fibroblast growth factor-activated receptor activity |  |  |  | 3xTg-AD mice treated with a non-specific activator of FGFR1 for 20 days (Murphy et al., 2018)  FGFR agonist in dopaminergic, hippocampal, and cerebellar granule rat neurons (Neiiendam et al., 2004)  FGFR agonist in Aβ_25-35_ injected adult rats (Enevoldsen et al., 2012) | 2.04 |
| Alcohol dehydrogenase | HPGD-15 | Acetaldehyde dehydrogenase activity |  |  | Protein increased (Shin et al., 2021) | Pharmacological inhibition of HGPD-15 in 6-month-old 5xFAD mice (Shin et al., 2021) | 1.36 |
| B6 | NPTX2 | Post-synaptic neurotransmitter receptor activity | Co-localizes with tau deposits (Xiao et al., 2017) | Transgene overexpression in primary rat cerebrocortical neuron cultures (Abad et al., 2006) | Protein increased in cortical neuronal cultures exposed to Aβ oligomers (prior to neurotoxicity) (Abad et al., 2006)  Protein increased in late-onset sporadic AD (Abad et al., 2006) and MCI, but decreases in later dementia stages (Duits et al., 2018) | Knockdown in cortical neuronal cultures exposed to Aβ oligomers (Abad et al., 2006) | 1.04 |
| Ascorbate ferrireductase | FRRS1 | Transmembrane ascorbate ferrireductase activity | AD-risk gene (Binder et al., 2022) |  | mRNA reduced/  Protein increased (Binder et al., 2022) |  | 0.91 |
| Lachesin | NEGR1 | Protein homodimerization activity | AD-risk gene (Ni et al., 2018) | *Negr1*^-/-^ mice (Singh et al., 2019) |  |  | 0.82 |
| SP1029 | ANPEP | Predicted to enable metalloamino-peptidase activity |  |  | mRNA increased in tau^P301L^ transgenic mouse cerebellum/  Protein increased in the frontal cortex of FTD patients compared to controls (Karsten et al., 2006) | Genetic overexpression suppresses tau^P301L^-induced neurodegeneration in *Drosophila* (Karsten et al., 2006) | 0.70 |
| Mt-ND2 | mt-ND2 | Predicted to enable NAD dehydrogenase activity | Changes expression in the presence of neurofibrillary tangles in AD brain (Cruz-Rivera et al., 2018) | ND2 mutant *Drosophila* (Burman et al., 2014) | mRNA reduced in human AD frontal cortex (Lee et al. 2018) |  | 0.61 |

AD = Alzheimer’s disease; MCI = Mild cognitive impairment; FGFR1/3 = fibroblast growth factor receptor 1/3; HPGD-15 = 15-hydroxyprostaglandin dehydrogenase; NPTX2 = neuronal pentraxin 2; FRRS1 = ferric chelate reductase 1; NEGR1 = neuronal growth regulator 1; ANPEP = alanyl aminopeptidase; mt-ND2 = mitochondrially encoded NADH:ubiquinone oxidoreductase core subunit 2.

**Supplemental Table 3 | Differentially translated mRNA in tau^R406W^ transgenic *Drosophila* have been implicated in neurodegenerative disorders.** Summary of the current literature findings linking the profiling-recognized, efficiently translated genes to Alzheimer’s disease and neurodegeneration.
